# Acute anterior uveitis after photorefractive keratectomy: Demographic profile and clinical characteristics

**DOI:** 10.22336/rjo.2025.09

**Published:** 2025

**Authors:** Ashish Markan, Shivani Chabbra, Mohammed Ibrahime Asif, Rahil Chaudhary, Manasi Tripathi

**Affiliations:** 1 Eye7 Chaudhary Eye Hospitals, New Delhi, India; 2 Dr. Rajendra Prasad Centre for Ophthalmic Sciences, AIIMS New Delhi, India

**Keywords:** uveitis, photorefractive keratectomy, complications, AAU = Acute anterior uveitis, PRK = Phototherapeutic keratectomy, UDVA = Uncorrected Distance Visual Acuity, CDVA = Corrected Distance Visual Acuity, MRSE = Mean Refractive Spherical Equivalent, IOP = Intraocular Pressure, RSBT = Residual Stromal Bed Thickness, PTA = Percentage Tissue Altered, SUN = Standardized Uveitis Nomenclature

## Abstract

**Objective:**

To report demographic profile and clinical characteristics of acute anterior uveitis (AAU) after Photorefractive Keratectomy (PRK).

**Materials and methods:**

This retrospective study reviewed records of all patients who underwent PRK to correct ametropia between July 2021 and June 2023. Patients who developed postoperative AAU were included for evaluation. Demographic details, preoperative ocular examination, intraoperative details, postoperative examination, time to onset of AAU, grading of cells, flare and pigments, time to heal, and final visual outcome were analyzed.

**Results:**

Records of 390 patients who underwent PRK during the study period were reviewed. Of these, 16 (29 eyes, b/l:13 patients, u/l: 3 patients) presented with AAU following PRK. The mean age of patients was 27.43 + 4.53 years (range 22-32 years), with a mean spherical equivalent of -3.18 + 2.16 (range -2 to -7 D). Mean ablation depth was 55.13 + 28.10 mm (range 27-105 mm), and mean duration of excimer laser ablation was 11.27 + 8.31 seconds (range 3-36 seconds). The mean onset time of AAU post-surgery was 27.8 + 10.9 days (range 7-47 days). Most eyes (75.86%, n=22) had a moderate-intense grade of inflammation, while 82.75% (n=24) of the eyes had significant pigment dispersion. The mean healing time was 57.43 + 27.87 days (33-106 days). The median follow-up duration was 12 months (range 6-18 months). The incidence of AAU post-PRK in our study was 4.1%.

**Discussion:**

This study’s incidence of post-PRK acute anterior uveitis (AAU) was 4.1%, aligning with previous reports of rare but significant inflammatory responses following PRK. The mean onset at 27.8 days suggested a delayed immune-mediated reaction rather than an immediate post-surgical response. While most cases had mild-to-moderate inflammation, a subset experienced severe reactions and ocular hypertension, reinforcing the need for close monitoring. The absence of systemic associations and posterior segment involvement suggested the role of a localized immune response. Despite the prolonged resolution time, all eyes achieved a final CDVA of 0 LogMAR, indicating favorable long-term outcomes with timely intervention and management.

**Conclusion:**

Anterior uveitis following PRK is infrequent. While it presents with marked anterior chamber reaction and pigment dispersion, the inflammation is often well-controlled with topical steroids. It does not affect the final visual outcome.

## Introduction

With the rise in the sophistication of technology in ophthalmology, the demand for achieving spectacle independence has significantly increased in recent years. Even in the latest advances in refractive surgery, photorefractive keratectomy (PRK) has stood the test of time. It remains a preferred option in many cases, such as subtle topographic abnormalities, thin corneas, or those undergoing re-surgery for residual refractive errors [1-4]. While challenges such as post-operative redness, discomfort, pain, abnormal epithelial remodeling, and potential haze persist [5], PRK delivers excellent visual outcomes with long-term stability [6].

Acute anterior uveitis is a rare but significant adverse event among potential complications [7]. This study aims to analyze and characterize the demographic profile and clinical features of post-PRK acute anterior uveitis.

## Materials and methods

In this retrospective case series, the medical records of all patients who underwent PRK at the study center were reviewed, and the details of patients who developed anterior uveitis after PRK were studied. Demographic data, including age at the time of surgery, sex, any systemic illness, and preoperative details such as mean refractive spherical equivalent (MRSE), corrected distance visual acuity (CDVA), intraocular pressure (IOP), anterior segment and posterior segment examination were noted. Corneal topography was captured for all patients on Pentacam^®^ (OCULUS, Germany) to study their corneal topography and assess eligibility for refractive surgery. Patients with mild-moderate myopia, with no ocular pathology other than refractive error, thinnest pachymetry of ≥ 470 microns, residual stromal bed thickness (RSBT) ≥ 300 microns and ≤ 40% percentage tissue altered (PTA) underwent alcohol assisted PRK under topical anesthesia.

### Surgical Procedure

Proparacaine 0.5%, a topical anesthetic agent, was instilled 5 minutes before the surgery. The epithelium was loosened using a 20% alcohol solution, followed by manually removing epithelium using a Merocel sponge/debrider. The optical zone diameter for all subjects was 6.5 mm. Corneal ablation was done using WaveLight® Excimer Laser. Mitomycin-C 0.02% was applied to the ablated cornea for 10 seconds per diopter, ensuring no contact was made with the limbus/conjunctiva. The area was then thoroughly irrigated with 20 ml cold balanced salt solution. A bandage contact lens (BCL) was placed at the end of the procedure. The post-operative medications included topical moxifloxacin 0.5% TDS for 2 weeks. Topical steroids (E/d Loteprednol 0.5% QID) were started on the same day and were slowly tapered over 3 months. Additionally, topical sodium hyaluronate 0.1% QID was prescribed for 3 months with oral analgesic for 3 days. BCL was removed 5 days after the surgery. Patients were followed up at POD-1, POD-7, 1 month, 3 months, and 6 months.

In the post-operative period, UCVA, IOP, time to onset of symptoms, characteristics of symptoms, anterior chamber evaluation including the grade of cells, flare and pigments (as per SUN classification [8]), and posterior segment evaluation were noted. A thorough systemic history was enquired, and a relevant physical examination was performed to rule out the systemic associations of anterior uveitis. All these patients also underwent testing for HLA-B27, ESR, CRP, Serum ACE, Viral Markers (HIV, Hepatitis B, Hepatitis C), Mantoux test, and Chest X-ray to ascertain the same. Statistical analysis was done using IBM SPSS software (version 16.0, IBM Corp.).

The study’s primary objective was to evaluate the presenting clinical features and grading of anterior chamber inflammation. The secondary objective was to study the time to onset, time to resolution, and final visual outcome regarding uncorrected distance visual acuity.

## Results

A total of 16 patients (29 eyes) were included. The mean age of the patients was 27.43 + 4.53 years (14 males, two females). None of the patients had any known systemic illness. The preoperative MRSE was -3.18 + 2.16 D, and all patients had CDVA 0 Logmar. The mean preoperative IOP was 17.4 ± 1.97 mmHg. Considering the intra-operative parameters, the mean ablation depth was 55.13 + 28.10 mm, and the mean duration of excimer laser ablation was 11.27 + 8.31 seconds. A summary of baseline parameters is provided in **Table 1**.

**Table 1:** Baseline preoperative parameters of patients

Age	27.43 + 4.53 years
**Male: Female**	7:1
**MRSE**	-3.18 + 2.16 D
**CDVA**	0 Log MAR (100%, n=29, eyes)
**IOP**	17.4 ± 1.97 mmHg
**Ablation Depth**	55.13 + 28.10 mm
**Ablation time**	11.27 + 8.31 seconds

The mean time to onset of acute anterior uveitis in the postoperative period was 27.8 + 10.9 days (7-47 days). All patients presented with complaints of redness (n=16 patients), and 62.5% (n=10 patients) had associated photophobia. On examination, 24.1% of eyes (n=7) had one line drop in UDVA, while 75.86% (n=22) maintained UDVA of 0 Log MAR.

On slit lamp examination of anterior chamber (AC), 20.6% (n=6) eyes had mild reaction (0.5+ AC cells), 34.48% (n=10) had moderate (1+ AC cells), 41.37% (n=12) had severe reaction (2+ to 3+ AC cells) while 3.4% (n=1) had an intense reaction (4+ AC cells). There was a weak positive correlation between the depth of ablation and the grade of inflammation. However, this correlation was not statistically significant (r=0.27; p-value >0.05). Additionally, 82.75% (n=24) of eyes had significant pigment dispersion. 89.65% (n=26) of the eyes had mild flare while 10.3% (n=3) had no flare. None of the eyes had posterior segment involvement (**Table 2**).

**Table 2:** Summary of characteristics of anterior uveitis

Time to onset	27.8 + 10.9 days		
**UDVA at presentation**	0.053 ± 0.08 LogMAR 75.8% (n=22) with UCVA 6/6 24.13% (n=7) with UCVA worse than 6/6		
**IOP at presentation**	14.21 ± 3.3 mmHg		
**Anterior chamber examination**	**Anterior chamber cells**		
	Mild (+0.5)	20.6% (n=6)	
	Moderate (+1)	34.48% (n=10)	
	Intense (+2 to +3)	41.37% (n=12)	
	Severe (+4)	3.4% (n=1)	

	Pigment Dispersion	82.75% (n=24)	
	Flare	No flare	10.3% (n=3)
		Mild Flare	89.65% (n=26)
**Posterior segment involvement**	No cases		
**Steroid responder**	41.37 % (n=12)		
**Time to resolution**	57.43 + 27.87 days		
**Recurrence**	10.34 % (n=3)		
**Final CDVA at resolution**	0 LogMAR (100% eyes; n=29)		

**Fig. 1:**
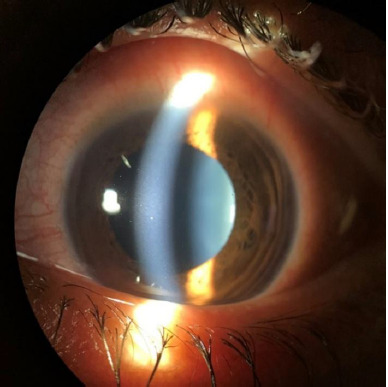
Anterior segment slit lamp photograph showing fine non-granulomatous KPs and anterior chamber cells

**Fig. 1** shows anterior segment slit lamp photograph highlighting fine non-granulomatous KPs and anterior chamber cells in a patient with AAU following PRK.

All patients were treated with topical prednisolone acetate (1%) and topical homatropine (2%), tailored according to the severity of inflammation and symptoms. The immunological profile was adverse in all our patients. 41.37% (n=12) of eyes developed ocular hypertension during treatment for anterior uveitis. All these cases were resolved upon tapering or cessation of topical steroids.

The mean time to heal was 57.43 + 27.87 days, ranging from 33 to 90 days. 11.53% (n=3) of eyes had recurrent uveitis. The mean follow-up duration was 4.2 + 1.56 months. At their last follow-up, 100% (n=29) of eyes had UDVA of 0 Log MAR. In the two years of our study, the incidence of AAU post-PRK was 4.1%.

## Discussion

Photorefractive keratectomy (PRK) is a laser refractive procedure that uses excimer laser to correct ametropia. Though not as widely popular as LASIK and emerging Kerato-lenticular Extraction or KLex surgeries, PRK is still preferred over the recent advances in cases with thin cornea, eyes with too high or too flat corneas or as a secondary enhancement procedure after previous refractive surgery [9], to manage residual refractive error post-cataract surgery [2] or to correct post-penetrating keratoplasty astigmatism [10].

Apart from the common issues of post-operative pain and corneal haze, acute anterior uveitis after PRK is among the rarely reported complications [7]. Even though PRK is essentially a surface procedure where the integrity of the anterior chamber is maintained, the excimer laser used for corneal ablation does alter the blood-aqueous barrier [11,12]. However, this breakdown has been reported to be transient, and the flare returns to baseline in the early post-op period. This transient increase in anterior chamber reaction is postulated to be associated with the activation of several chemical inflammatory mediators [13].

In contrast to previous studies, our patients had a delayed onset of anterior uveitis and were highly symptomatic. Our results are consistent with those of Dhami et al. [7], who also reported a delayed onset of AAU after TransPRK. The cause of the delay in these subsets of patients is unclear. One possible hypothesis is a delayed hypersensitivity reaction to excimer laser-induced changes in the corneal microstructure, which could be related to the different ethnicities of these patients compared to previous studies [14].

Like the results in a study by Dhami et al., another point to highlight here is that our patients underwent alcohol-assisted epithelium debridement before excimer laser compared to single-step Trans-PRK. Despite this difference, the incidence and time to develop uveitis during the postoperative period were similar in both studies [7].

The reported incidence of postoperative uveitis following LASIK surgery is 0.06-0.18% [15,16]. This is far less than what has been seen in patients undergoing PRK. Studies have shown an increased pro-inflammatory cytokine release after PRK compared to LASIK from the stimulated corneal fibroblasts [17,18]. An exaggerated inflammatory response in PRK could explain an increased incidence of uveitis in PRK compared to LASIK.

None of the patients in our study exhibited any signs of underlying systemic disease. All underwent comprehensive blood tests, including screening for HLA-B27, and all tested negative. Similarly, Dhami et al. found no systemic association for uveitis in their cohort. Thus, AAU following PRK appears to be independent of systemic association and is primarily iatrogenic, likely related to surgical trauma [19,20].

Building on existing literature, our study provides further insights into the clinical characteristics of AAU associated with PRK. PRK-associated anterior uveitis tends to have intense anterior chamber inflammation with significant pigment dispersion. All cases were easily managed with an intensive topical steroid-cycloplegic combination. Most cases had a single episode of uveitis over a one-year follow-up. Only three patients developed a recurrent attack, which was again managed with a short course of topical steroid-cycloplegic combination. Lastly, uveitis did not impact the overall visual outcome post-refractive surgery; all patients ultimately achieved an uncorrected distance visual acuity (UDVA) of 0 Log MAR after resolving the inflammatory episode.

One limitation of our study was its retrospective design, which inherently limited the ability to establish causality and introduced potential biases. Future prospective, long-term studies are needed to provide a more comprehensive understanding of AAU following PRK, allowing one to observe patterns, risk factors, and outcomes in real time. Additionally, our study did not include objective quantification of anterior chamber inflammation. Utilizing tools like a laser flare meter for the quantitative assessment of cells and flare could have yielded more precise data, allowing for stronger correlations and a deeper understanding of the inflammatory process in this patient population.

## Conclusion

To conclude, acute anterior uveitis following PRK is a rare event. Although the cases in our study were characterized by moderate to severe inflammation, they resolved with topical steroid therapy alone. The AAU episode did not alter the final visual outcome after refractive surgery.
